# Acute stress affects risk taking but not ambiguity aversion

**DOI:** 10.3389/fnins.2014.00082

**Published:** 2014-05-06

**Authors:** Magdalena Buckert, Christiane Schwieren, Brigitte M. Kudielka, Christian J. Fiebach

**Affiliations:** ^1^Department of Psychology, Goethe University Frankfurt am MainFrankfurt am Main, Germany; ^2^Department of Economics, University of HeidelbergHeidelberg, Germany; ^3^Department of Psychology, University of RegensburgRegensburg, Germany; ^4^IDeA Center for Individual Development and Adaptive Education, Goethe University Frankfurt am MainFrankfurt am Main, Germany; ^5^Donders Center for Cognition, Donders Institute for Brain, Cognition and Behaviour, Radboud University NijmegenNijmegen, Netherlands

**Keywords:** stress, cortisol, risk, ambiguity, uncertainty, gain/loss domain, decision making

## Abstract

Economic decisions are often made in stressful situations (e.g., at the trading floor), but the effects of stress on economic decision making have not been systematically investigated so far. The present study examines how acute stress influences economic decision making under uncertainty (risk and ambiguity) using financially incentivized lotteries. We varied the domain of decision making as well as the expected value of the risky prospect. Importantly, no feedback was provided to investigate risk taking and ambiguity aversion independent from learning processes. In a sample of 75 healthy young participants, 55 of whom underwent a stress induction protocol (Trier Social Stress Test for Groups), we observed more risk seeking for gains. This effect was restricted to a subgroup of participants that showed a robust cortisol response to acute stress (*n* = 26). Gambling under ambiguity, in contrast to gambling under risk, was not influenced by the cortisol response to stress. These results show that acute psychosocial stress affects economic decision making under risk, independent of learning processes. Our results further point to the importance of cortisol as a mediator of this effect.

## Introduction

Stress is an increasingly important factor influencing our daily lives. It primarily activates two systems: the sympathetic nervous system (SNS) and the hypothalamus-pituitary-adrenal (HPA) axis (e.g., Kudielka and Kirschbaum, 2007). Heart rate can be taken as a proxy for sympathetic activity, whereas the adrenal hormone cortisol indicates the activity of the HPA axis. Cortisol is secreted into the blood stream by the adrenal glands in response to the hormonal cascade of the HPA axis. As a steroid hormone, cortisol crosses the blood-brain barrier, and thereby reaches the brain. Cortisol binds to two types of receptors, with high affinity to mineralocorticoid receptors and with lower affinity, i.e., only if concentration is high as for example under acute stress, to glucocorticoid receptors (De Kloet, 2004). The latter receptors are ubiquitously expressed in the brain, so cortisol can exert diverse modulating influences on emotional as well as cognitive processes.

The influence of stress on cognition is well documented for example in the domain of long-term memory (Wolf, 2009). Recent studies also investigated other cognitive functions like working memory (e.g., Schoofs et al., 2008; see also Buckert et al., 2012) and executive functions (e.g., Scholz et al., 2009; Plessow et al., 2012) under acute stress. Much less research exists concerning the influence of stress on decision making, even though many decisions—particularly in the economic area—are made under stress, be it time pressure, work load, pressure of competition, or threat of job loss. It is thus of great importance to investigate the effect of stress on decision making. While recent studies indeed provide first evidence that decision making is influenced by acute stress (for a recent review see Starcke and Brand, 2012), the specific mechanisms of how stress influences decision making are not yet understood.

Most studies report more risk taking under acute stress (Preston et al., 2007; Starcke et al., 2008; Lighthall et al., 2009; van den Bos et al., 2009; Pabst et al., 2013c). Yet, one study found increased risk taking only for losses, while for gains risk taking was decreased by stress (Porcelli and Delgado, 2009). Still another study reported less risk taking only for losses whereas for gains stress had no effect (Pabst et al., 2013b). Clark et al. (2012) observed risk avoidant behavior in trials containing losses when the threat of an electric shock was present. Some studies also found no differences in risk taking between stress and control groups (Lempert et al., 2012; Gathmann et al., 2014). Furthermore, some of the studies observed gender interactions with stress, i.e., stressed men being risk seeking and stressed women being risk avoidant (Preston et al., 2007; Lighthall et al., 2009) unless very high levels of cortisol are reached (van den Bos et al., 2009). However, others did not find any gender differences in decision making under stress (Starcke et al., 2008; Pabst et al., 2013b,c). Related studies investigating risk taking under time pressure found that risk taking was differentially affected for gains, losses, and mixed options (Ben Zur and Breznitz, 1981; Jones et al., 2011; Young et al., 2012; Kocher et al., 2013). Taken together, it can thus be concluded that stress effects on risk taking are plausible, but their exact nature and the conditions under which stress influences risk taking are only beginning to be understood.

Previous studies differ regarding several aspects that might be responsible for the somewhat mixed results reported (Table 1). First of all, different stressors might account for some heterogeneity. Several studies applied psychosocial stress induction procedures (Preston et al., 2007; Starcke et al., 2008; van den Bos et al., 2009; Pabst et al., 2013a,b,c; Gathmann et al., 2014), others used physiological stressors (Lighthall et al., 2009, 2012; Porcelli and Delgado, 2009), still others the threat of an electric shock (Clark et al., 2012). Time pressure could also be seen as a stressor. Yet, physiological parameters have rarely been assessed in time pressure studies, thus making a direct comparison with other stressors difficult. It is conceivable that the different stressors evoked diverging patterns of physiological and neuronal activity, thereby leading to different effects on decision making. A recent study also pointed to the importance of the timing of the decision making task in relation to the stress induction (Pabst et al., 2013a). Whereas choice behavior was risk avoidant 5 and 18 min. after stress onset, more risk taking was observed 28 min. later when cortisol levels peaked.

**Table 1:** **Previous studies of stress effects on decision making under uncertainty**.

**Study**	**Task**	**Domain**	**Stressor**
Preston et al. (2007)	IGT (ambiguity)	mixed	Anticipatory (speech)
van den Bos et al. (2009)	IGT (ambiguity)	mixed	TSST
Lighthall et al. (2009)	BART (ambiguity)	gain	Cold pressure
Lighthall et al. (2012)	BART (ambiguity)	gain	Cold pressure
Young et al. (2012)	Bets (ambiguity, risk)	gain	Time pressure
Starcke et al. (2008)	GDT (risk)	mixed	Anticipatory (speech)
Pabst et al. (2013a)	GDT (risk)	mixed	TSST
Pabst et al. (2013b)	GDT (risk)	gain, loss	TSST
Pabst et al. (2013c)	GDT (risk)	mixed	TSST
Gathmann et al. (2014)	GDT (risk)	mixed	TSST
Ben Zur and Breznitz (1981)	Lotteries (risk)	mixed	Time pressure
Porcelli and Delgado (2009)	Lotteries (risk)	gain, loss	Cold pressure
Jones et al. (2011)	Lotteries (risk)	mixed	Time pressure
Clark et al. (2012)	Lotteries (risk)	mixed, gain, loss	Threat of electric shock
Lempert et al. (2012)	Lotteries (risk)	gain	Anticipatory (speech)
Kocher et al. (2013)	Lotteries (risk)	mixed, gain, loss	Time pressure

Previous studies differed regarding the stressor employed (TSST, Trier Social Stress Test), the decision making task (IGT, Iowa Gambling Task, BART, Balloon Analogue Risk Task, GDT, Game of Dice Task), as well as the domain of decision making (mixed: contains gains and losses). If probabilities are given, the task is considered to assess risk, whereas if probabilities are not provided, decisions are considered to be made under ambiguity.

Furthermore, when studying decision making under uncertainty, risk can be differentiated from ambiguity (e.g., Knight, 1921/2012; Ellsberg, 1961; Brand et al., 2006; Weber and Johnson, 2009)^1^. Most decisions we usually face contain some degree of uncertainty, which means that the outcome of the decision is not certain beforehand. Uncertainty can be thought of as a continuum from certainty to complete ignorance (Starcke and Brand, 2012). In that context, risk means that while the actual outcome of a decision is unknown, the probabilities for each possible event are known to the decision-maker. In contrast, in ambiguous decisions, information regarding probabilities is lacking, either in part or completely (Starcke and Brand, 2012). Related to this distinction is the discrimination between decisions from description vs. decisions from experience (Hertwig and Erev, 2009). While the features of risky decisions can easily be described explicitly, aspects of ambiguous decisions like probabilities often need to be acquired through experience, i.e., learning from feedback.

In previous studies of stress effects on decision making, the distinction between risk and ambiguity has not been explored systematically. Preston et al. (2007) and van den Bos et al. (2009) used the well-known Iowa Gambling Task (IGT; Bechara et al., 1994). Lighthall et al. (2009) applied the Balloon Analogue Risk Task (BART; Lejuez et al., 2002). For both games, the probability distribution is not known to the subjects beforehand but has to be acquired through learning (IGT) or is dynamically changing (BART), respectively. Therefore, decisions in these tasks have to be made under ambiguity (cf. Fecteau et al., 2007b; Lighthall et al., 2009).

Risk taking was assessed either using lotteries (Porcelli and Delgado, 2009; Lempert et al., 2012), a common form of presenting choices in behavioral economics, or the so-called Game of Dice Task (GDT; Brand et al., 2005; Starcke et al., 2008; Pabst et al., 2013a,b,c; Gathmann et al., 2014). Nevertheless, the results from the latter studies are somewhat inconsistent in so far as some observed more risk taking under acute stress (Starcke et al., 2008; Pabst et al., 2013c; but see Lempert et al., 2012; Gathmann et al., 2014) while other studies reported differentially affected risk taking for gains and losses (Porcelli and Delgado, 2009; Pabst et al., 2013b).

It is difficult to draw definite conclusions from those studies as they differ in several further aspects, most importantly the decision making domain. Here, pure domains,—i.e., options that contain only positive (pure gain) or only negative (pure loss) amounts—are distinguished from the mixed domain in which one option can yield either a gain or a loss, and it is known that the domain can influence decision making (e.g., Kahneman and Tversky, 1979). Whereas pure gain and pure loss domains were used in the lotteries of Porcelli and Delgado (2009) and the modified GDT of Pabst et al. (2013b), the IGT (Preston et al., 2007; van den Bos et al., 2009) and the original GDT (Starcke et al., 2008; Pabst et al., 2013a,c) provide mixed domain options. Therefore, the possibility exists that risk taking is differentially influenced by stress depending on the decision making domain.

Furthermore, expected value was not systematically varied in the above-cited studies of stress effects on decision making. In some games, like the IGT (Bechara et al., 1994) and the GDT (Brand et al., 2005), the riskier options always have lower expected values (and are therefore disadvantageous), whereas in the lotteries used by Porcelli and Delgado (2009), expected values were kept constant across all options. In the BART (Lejuez et al., 2002), the expected value is changing with each pump of the balloon.

Besides the differences reported so far, almost all of the previously described studies share one important task feature, i.e., they provided feedback about the outcome immediately after each choice. This enables learning processes and, indeed, disturbed learning from feedback seems to be one very plausible mechanism by which stress exerts its detrimental effect on decision making (Petzold et al., 2010; Mather and Lighthall, 2012). Yet, by providing feedback the distinction between decisions from description and decisions from experience (see above) can no longer be made. Therefore, it is unclear if feedback learning is the only process affected by stress or if stress also exerts effects on risk taking as such, i.e., without feedback. Empirical evidence is inconclusive so far as one study that elicited indifference points between risky options and sure amounts did not provide feedback and found no stress effect (Lempert et al., 2012). Yet, another study also included an experimental condition without feedback (Starcke et al., 2008). For this condition, the authors still observed a group difference between stress and control groups that was significant at trend level (*p* = 0.08).

To summarize, up to now a definite answer is lacking for the question if risk taking *per se* is affected by stress. In case this can be confirmed, then the potential impact of the type of uncertainty and the decision domains warrants further investigation. Therefore, our study was designed to investigate these questions systematically by applying a decision making task that distinguishes risk from ambiguity, that varies domains and expected value orthogonally, and that does not provide feedback.

## Materials and methods

### Sample

The study procedure was approved by the ethics committee of the German Psychological Society. Eighty-nine participants, mostly students of the University of Heidelberg, took part in the study after giving written informed consent. Eight subjects had to be excluded from analysis as they met one of our exclusion criteria (i.e., mental, physical, or neurological disorders, intake of medication^2^, use of illicit drugs or heavy smoking, as well as a native language other than German). Furthermore, five subjects could not be included in the analysis because of problems with data collection. One subject in the control group showed a marked cortisol response (i.e., above our responder criterion of 2.5 nmol/l, see below) and was therefore also excluded. The remaining sample consisted of 75 participants (39 women and 36 men). Mean age of the sample was 21.95 ± 2.76^3^, mean BMI was 22.14 ± 2.53.

Participants were randomly assigned to either the stress (55 participants) or the control condition (20 participants). We recruited more subjects for the stress condition as we observed a very high rate of nonresponders (*N* = 29) to the TSST-G procedure during data collection (see below).

### General procedure

All sessions started at 14:30 h to minimize confounding influences of the cortisol diurnal rhythm, and lasted 2 h. Participants were requested to abstain from eating, drinking, smoking, or exercising within 1 h prior to the study. After arriving at the laboratory, participants were equipped with heart rate measurement devices (see below) and filled in questionnaires on demographic data and personality characteristics. After half an hour, mild psychosocial stress was induced using the Trier Social Stress Test for Groups (TSST-G) in the stress group, whereas the control group went through a control procedure, respectively (von Dawans et al., 2011). Immediately afterwards, participants worked on the economic decision making task for about 15 min. Then, further questionnaires were administered and working memory performance was assessed. During the experiment, seven saliva samples were collected for the analysis of cortisol (see below for details).

### Trier social stress test for groups (TSST-G)

For stress induction, a modified version (cf. Buckert et al., 2012) of the TSST-G (von Dawans et al., 2011) was applied in groups of four participants (in one session only three) of the same sex. Analogous to the original TSST (Kirschbaum et al., 1993), the paradigm was performed in a separate room and consisted of three stages: the preparation period, a free speech (8 min. total, i.e., 2 min. per participant), and a mental arithmetic task (8 min. total, i.e., 2 min. per participant). During the free speech, participants had to present themselves and argue why they believed to be the best candidate for their respective dream job. The mental arithmetic task consisted of counting backwards in two-digit steps starting with a four-digit number (e.g., counting backwards in steps of 17 starting with 2034). All tasks were performed in front of a white-coated jury consisting of a man and a woman, who stayed neutral throughout the test and did not give any social feedback. Additionally, the participants were videotaped with two digital camcorders. To maximize uncontrollability, the order in which participants had to perform the free speech and the mental arithmetic task was randomized. Further details about the setting can be found in von Dawans et al. (2011).

As control procedure, the placebo TSST-G described in von Dawans et al. (2011) was adapted and slightly modified. It consisted of a silent reading period (11 min.) and a paper-and-pencil mental arithmetic task (8 min.) in which arithmetic addition and subtraction problems of varying difficulty were handed out and participants were free to choose which one they wanted to work on and how many problems they wanted to solve. Participants were told that their results would not be controlled. The control procedure was performed in the same room as the TSST-G, but no videotaping was performed and no jury members were present. Instead, the experimenter stayed in the room to instruct the participants. Both procedures lasted approximately half an hour.

### Decision making task

The decision making task was programmed in z-tree (U. Fischbacher, University of Zürich, http://www.iew.uzh.ch/ztree) and consisted of 90 binary lottery trials. In each trial, the participant was asked to choose between options A and B via mouse click. No feedback was provided. The task consisted of two parts, one investigating decision making under risk, the other investigating decision making under ambiguity. All trials were presented in randomized order.

**Figure 1:**
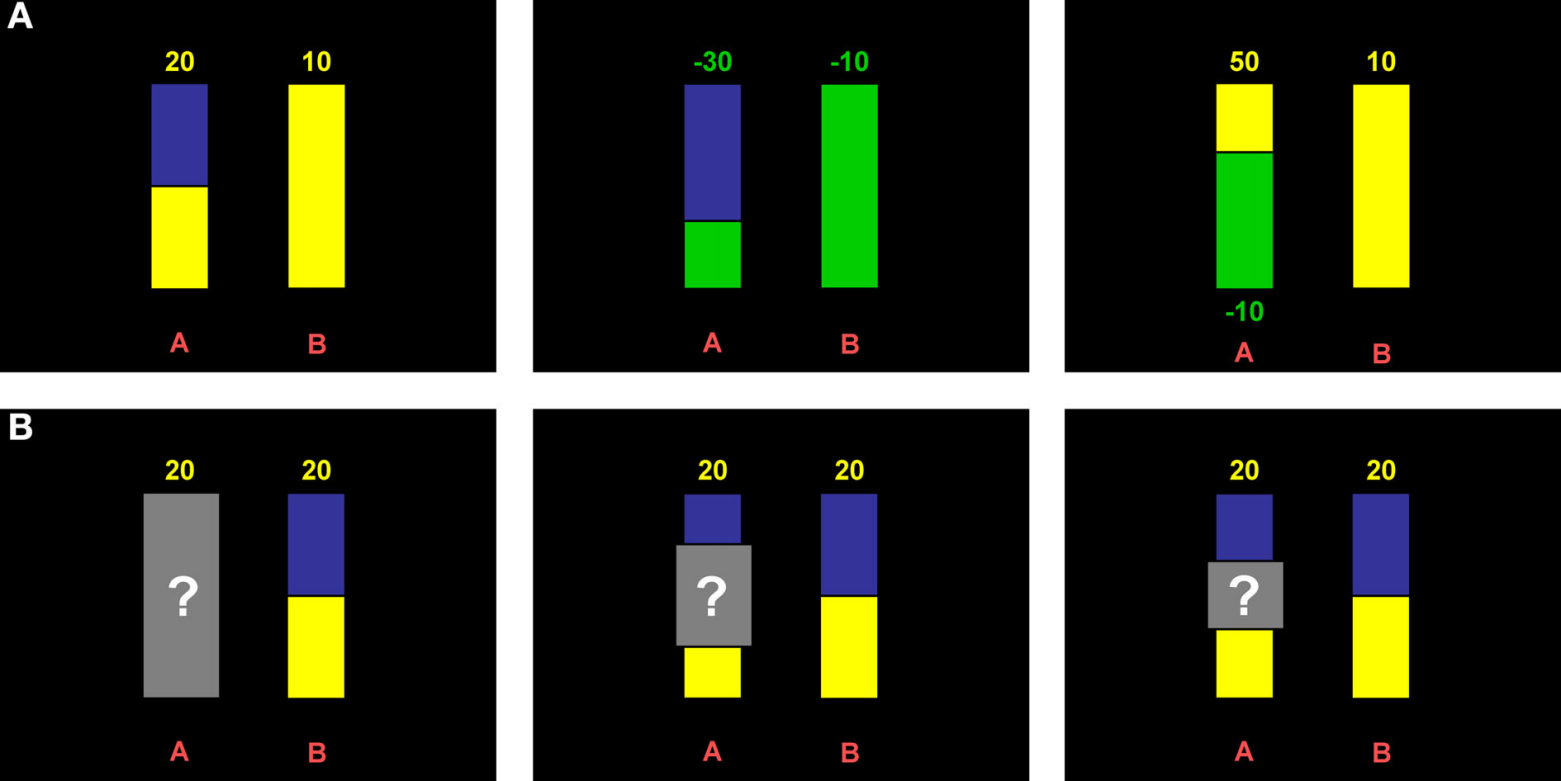
**Behavioral paradigm. (A)** Example displays of risk trials. Colors indicate the domains (yellow: gains; green: losses; blue: neutral). Amounts that can be won or lost are depicted above the bars, or in case of the mixed domain, above and below, respectively. Height of the colored part of the bars indicates the probability to win or lose and was varied at three levels (1/3, 1/2, 2/3). **(B)** Example displays of ambiguity trials. Ambiguity was introduced through gray occluders overshadowing all or part of the probability information. Occluder sizes varied at three levels (complete, 1/2, 1/3; cf. panels from left to right).

In the risk part, probabilities were displayed graphically as vertical bars similar to the display used by Hayden et al. (2010) and Putman et al. (2010). Pure gain, pure loss, and mixed decision domains were incorporated through color coding, i.e., yellow indicated winning and green indicated loosing while blue was the neutral color that was used for nonwinning or nonlosing in the pure domains (see Figure 1A). Potential reward values (in points that were later converted into money; see below) were displayed numerically above the bars and contained a minus sign for losses. In the mixed domain, gains were displayed above the bar and losses below the bar. The alternative option was always a sure gain (for pure gain and mixed domains) or a sure loss (for the pure loss domain) of 10 points, represented by a completely yellow or green bar, respectively. The position of the sure option (i.e., option A or B) was counterbalanced across participants.

The expected value of the risky option was varied at three levels, i.e., being higher, equal, or lower as compared to the sure amount. For the pure domains, this was accomplished through the combination of three reward values (15, 20, 30 points) and three probability levels (1/3, 1/2, 2/3), resulting in nine different items per domain (three items per level of expected value, i.e., higher vs. equal vs. lower). Each of these items was presented twice. In the mixed domain, either the gain or the loss amount was varied for a given probability level (again 1/3, 1/2, 2/3), yielding six different items per level of expected value. Each of the decision domains thus comprised 18 trials, i.e., 54 in total.

In the ambiguity part, ambiguity was introduced by gray occluders (cf. Hayden et al., 2010) equipped with a question mark overshadowing information about probabilities (Figure 1B). Three different occluder sizes (i.e., 1/3, 1/2, or complete) represented three different degrees of ambiguity. In this part, the alternative option was a risky lottery with a constant probability level of 0.5 and a constant reward value of 20 points. The occluders in the ambiguous option were centered around the probability level of the risky option, i.e., 50%. Again, expected value was varied at three levels analogous to the risk part by varying the reward values of the ambiguous option (i.e., 15, 20, 30 points). Only pure gain and loss domains were implemented (nine different trials each, three per expected value level) and all trials were presented once. Importantly, to overcome beliefs (see Trautmann and van de Kuilen, in press, for the important role of beliefs in ambiguity aversion) the instruction made explicit that all probabilities in the covered area are equally probable and that the actual probability is unknown also to the experimenter because it is determined by a computer program. Note that here ambiguity is operationalized as “second order probabilities” as opposed to the classic operationalization via Ellsberg urns (cf. Trautmann and van de Kuilen, in press).

As the ambiguity part differed from the risk part in several respects, we added nine trials to control for potential influences of the type of the alternative (i.e., risky lottery vs. sure amount) and the probability level (i.e., 1/3, 1/2, 2/3). These items were presented once in the gain and in the loss domain. Analysis of these additional items, however, gave no indication of confounding effects. We will thus not report results related to these items.

### Payment scheme

Participants received a small show-up fee (3 Euros). In the decision making task, they were instructed that one of the trials in each domain (pure gain: risk or ambiguity; pure loss: risk or ambiguity; mixed: risk) would be selected randomly and paid out. The points they won or lost in the selected three trials would be summed first and then converted into Euro by division of 2. If the sum was negative, nothing was paid, but subjects did not have to pay either. This was not told participants beforehand and no one asked about this possibility. Therefore, losses are supposed to be experienced as real losses during decision making (cf. Kocher et al., 2013). Participants received the money at the end of the experiment.

### Stress measures

#### Heart rate

Heart rate was measured continuously using Polar Sport Tester RS800CX (Polar Elektro GmbH, Groß-Gerau, Germany). Each participant was equipped with a breast belt and a display similar to a wrist watch. For analysis, the Polar Pro Trainer software was used. Data were aggregated into six intervals: pre-stress (duration: 15 min.), TSST-preparation (3 min.), TSST-speech (8 min.), TSST-arithmetic (8 min.), decision making task (10–15 min.) and post-stress (15 min.). Note that because of measurement failures in 11 persons, sample size is *N* = 64 for heart rate analysis.

#### Cortisol

Saliva samples were collected at seven time points throughout the experiment using Salivette collection devices (Sarsted, Nuembrecht, Germany): 15 min. after arriving (−15), 1 min. before (−1) and directly after the TSST-G (+1), as well as repeatedly afterwards (+15, +25, +45, +60 min.). For one subject, sample two was not utilizable, so for calculation of the variable cortisol increase (see below) sample one was used instead of sample two. For four other subjects, one of the other samples (samples 1, 6, or 7) could not be analyzed. Therefore, these five subjects are not included in the repeated measures analyses of cortisol data. Samples were analyzed by the Psychobiological Research Laboratory of the University of Trier, Germany, using a time-resolved immunoassay with fluorometric detection (DELFIA, cf. Dressendörfer et al., 1992). Intra-assay variation was 4.0–6.7%, inter-assay variation 7.1–9.0%.

#### Subjective mood rating

Subjective mood was assessed by the German mood questionnaire “*Mehrdimensionaler Befindlichkeitsfragebogen*” (MDBF; Steyer et al., 1997) before and directly after the TSST-G or control procedure, respectively. The MDBF consists of a list of adjectives constituting three scales (elevated vs. depressed mood, calmness vs. restlessness, wakefulness vs. sleepiness). Because of missing data, analyses are based on *N* = 73 for the mood and wakefulness subscales and *N* = 72 for the calmness subscale.

### Working memory

As several studies pointed to the fact that risk taking and working memory performance are related (Cokely, 2009; Corbin, 2010; Starcke et al., 2011; Brevers et al., 2012), we decided to include a short measure of working memory performance in our study ^4^. We used the digit span (forward) subtest of the German adaptation of the Wechsler Intelligence Scales for Adults (Tewes and Wechsler, 1991). This test was slightly modified for the use in a group setting. Subjects were asked to write down each memorized series of digits, rather than speaking them out loudly after listening to the experimenter reading them. Working memory was assessed in the resting phase after the decision making task (i.e., half an hour after cessation of the TSST-G or control procedure, respectively).

### Statistical analysis

Statistical analyses were carried out using the SPSS statistical software package (IBM SPSS Statistics, version 20; Chicago, IL, USA). The stress response was analyzed using repeated measures analyses of variance (ANOVAs) for heart rate (six time points) and cortisol data (seven time points). For cortisol data, log-transformed variables were used for statistical analyses. For better interpretability, untransformed raw data are displayed in the Figures. Potential confounding variables like age and BMI, as described in the literature, did not emerge any effects on cortisol and heart rate responses, and were therefore not included as covariates in the statistical analysis. Gender was always included as between-subjects factor. As we observed considerable variability in the cortisol response in the stress group, this group was split into cortisol responders and nonresponders by applying the literature-based criterion of an individual peak cortisol increase of 2.5 nmol/l or more which is thought to reflect a secretory episode (cf. Kirschbaum et al., 1992; Schommer et al., 2003). For all following analyses, three groups were compared, i.e., cortisol responders vs. nonresponders vs. nonstressed controls.

**Figure 2:**
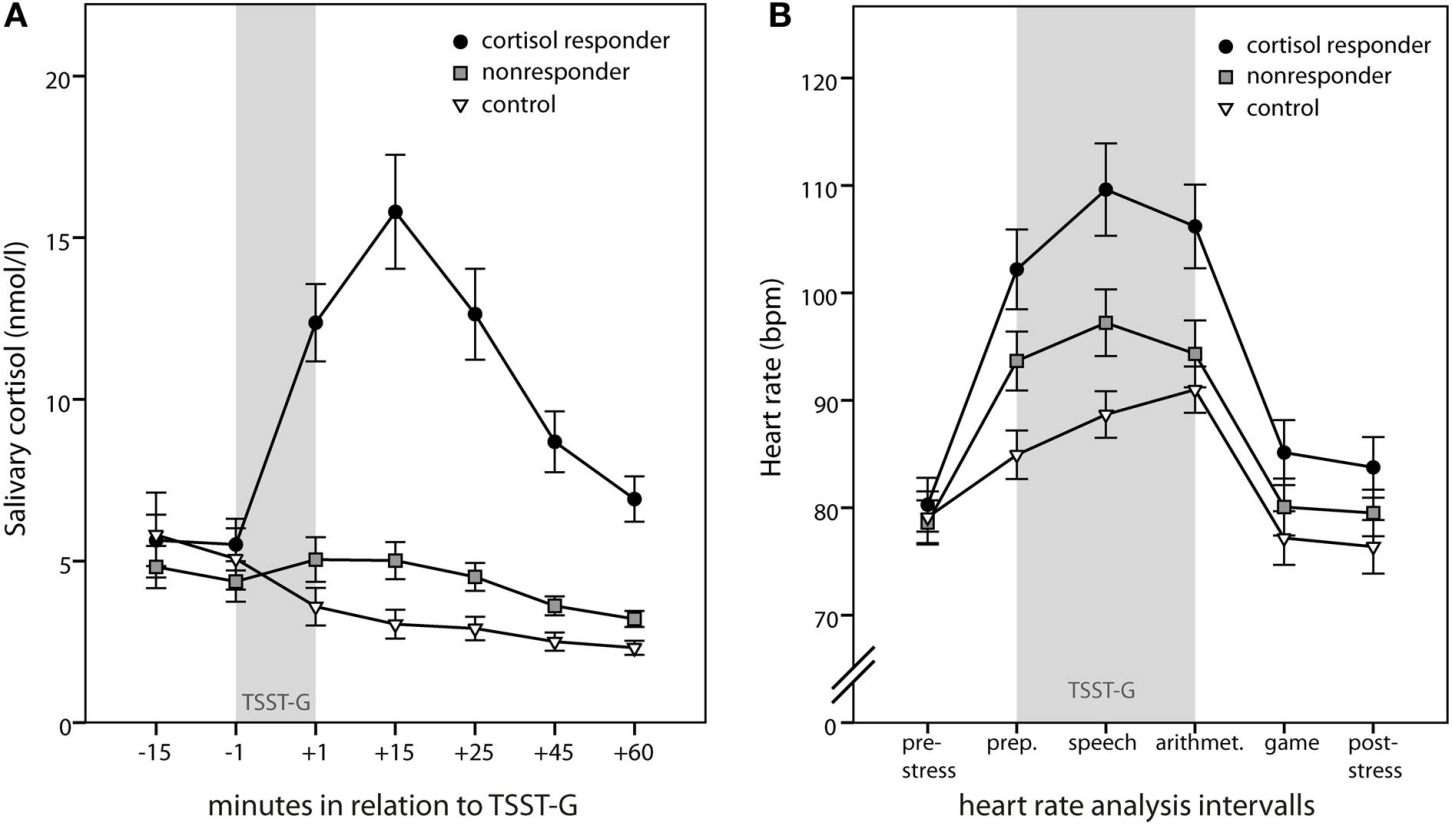
**Physiologic stress markers**. Time course of salivary cortisol **(A)** and heart rate **(B)**. Gray bars indicate the timing of the Trier Social Stress Test for Groups (TSST-G). Depicted are means ± standard errors of the mean (s.e.m.).

The individual peak cortisol increase was determined for each participant by subtracting the pre-stress cortisol level (measurement point −1; see Figure 2A) from the highest level reached afterwards (not log-transformed). Similarly, increase in heart rate was defined as the difference between the period with the highest mean heart rate during stress induction (i.e., preparation, speech, or arithmetic) and the pre-stress period. For subjective mood changes, difference scores between the pre- and post-stress measures for each MDBF scale were calculated. Group comparisons were performed using univariate ANOVAs including gender as additional factor.

Analysis of decision making behavior was done separately for the risk and the ambiguity part via repeated measures ANOVAs. Percentage of choices of the risky or ambiguous alternative, respectively, was the dependent variable in all analyses. Working memory performance was tested as covariate and included if significant influences were obtained. For the analysis of the risk part, the repeated measures ANOVA contained the within-subjects factors domain (gain, loss, mixed) and expected value of the risky alternative (higher, equal, lower) and the between-subjects factors group (cortisol responders, nonresponders, control) and gender. The same ANOVA was performed for the ambiguity part, except that here the factor domain had only two levels (gain, loss).

In all repeated measures analyses, Huynh-Feldt corrections were applied if sphericity was violated. Simple contrasts comparing cortisol responders to nonresponders and controls were used to follow up significant main effects in case of a priori hypotheses. Otherwise pairwise comparisons with Bonferroni corrected levels of significance were applied. Descriptive data are given as mean ± standard deviation.

## Results

### Stress induction

As groups were built upon the criterion of a peak cortisol increase equal or above 2.5 nmol/l, peak maximal cortisol increase was significantly different between the three groups [*F*_(2, 69)_ = 54.93; *p* < 0.001; 10.48 ± 6.45 nmol/l in responders vs. 1.01 ± 1.09 nmol/l in nonresponders and −1.22 ± 1.99 nmol/l in controls; both contrasts *p* < 0.001]. Gender differences were more pronounced in the cortisol responder and the control group compared to the nonresponder group [interaction group × gender: *F*_(2, 69)_ = 3.21; *p* = 0.047]; yet, gender effects per group were not significant. When including time, a significant group × time interaction [*F*_(4.7, 150.41)_ = 37.97; *p* < 0.001] emerged in the repeated measures analysis additionally to the main effect of group [*F*_(2, 64)_ = 23.34; *p* < 0.001], indicating that cortisol responders differed from nonresponders and controls specifically for the measurement points 3 through 7 (all *p* < 0.001) (Figure 2A). Furthermore, men had higher cortisol levels compared to women [*F*_(1, 64)_ = 5.59; *p* = 0.021], specifically at the first three measurement points [interaction time × gender: *F*_(2.35, 150.41)_ = 5.66; *p* = 0.003].

Similarly, peak heart rate increase differed significantly between groups [*F*_(2, 58)_ = 19.88, *p* < 0.001; 33.05 ± 12.58 bpm in cortisol responders vs. 20.30 ± 9.54 bpm in nonresponders and 12.19 ± 5.18 bpm in controls; both contrasts *p* < 0.001]. Again, this was also true for repeated measures analysis [main effect of group: *F*_(2, 58)_ = 4.28; *p* = 0.019; interaction time × group: *F*_(6.78, 196.58)_ = 7.50; *p* < 0.001]. Separate univariate ANOVAs for each measurement interval revealed that groups differed significantly only during the TSST-G (i.e., preparation, speech, and arithmetic; all *p* < 0.008). Simple contrasts confirmed that cortisol responders differed from nonresponders (all *p* < 0.026) as well as controls (all *p* < 0.006) during the TSST-G intervals (Figure 2B).

**Figure 3:**
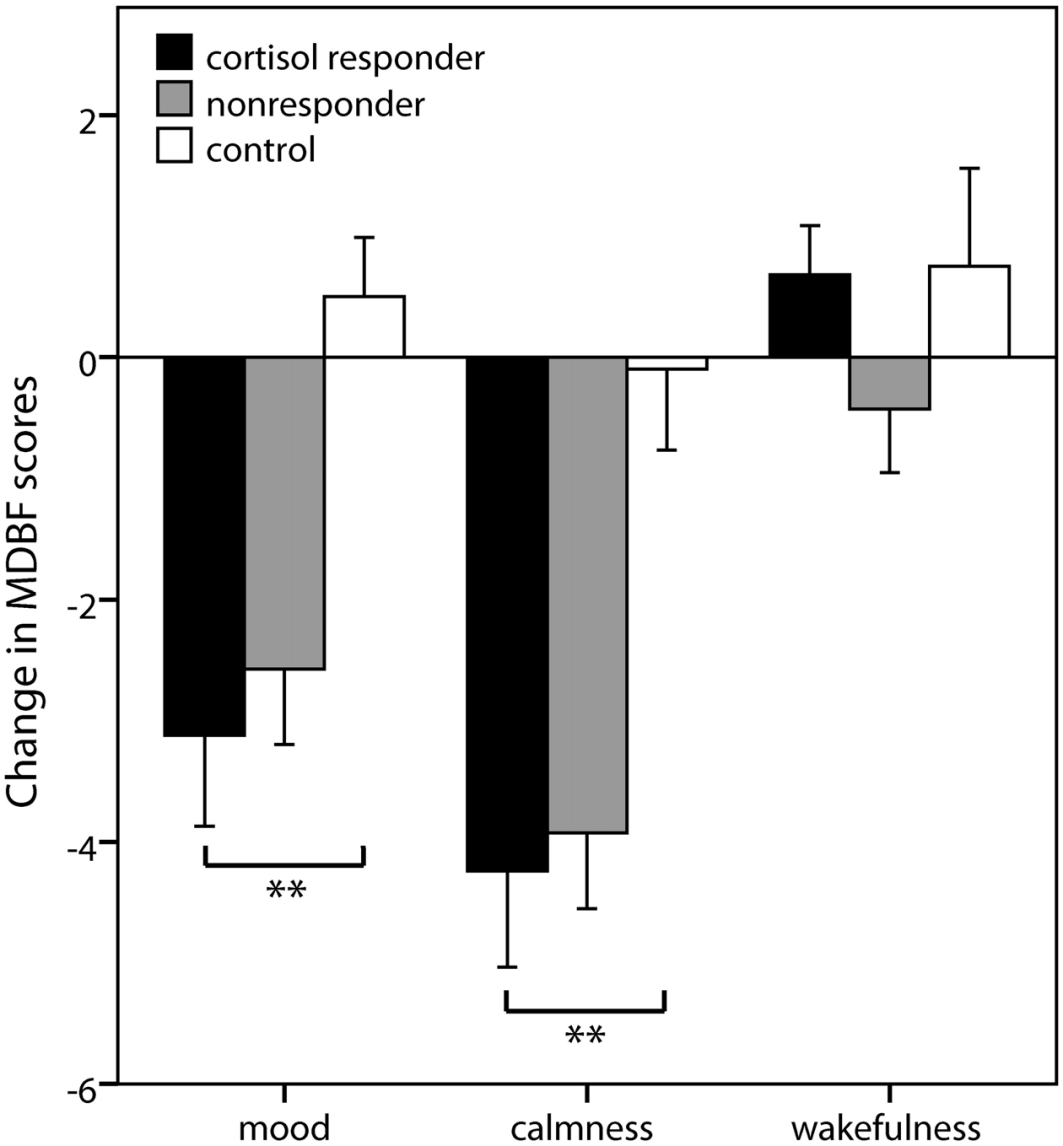
**Subjective stress markers**. Means ± s.e.m. of the change scores of the mood questionnaire MDBF (*Mehrdimensionaler Befindlichkeitsfragebogen*) from the measurement before the stress induction to the measurement afterwards. Negative change scores indicate an increase in negative mood (mood scale), an increase in restlessness (calmness scale), and an increase in sleepiness (wakefulness scale). Simple contrasts comparing cortisol responders to nonresponders and controls were used to follow up significant main effects (^**^p < 0.01).

Group differences in subjective changes were observed for mood [*F*_(2, 67)_ = 7.97; *p* = 0.001] and calmness [*F*_(2, 66)_ = 8.78; *p* < 0.001] ratings, but not for wakefulness ratings [*F*_(2, 67)_ = 1.31; *p* = 0.276]. Simple contrasts revealed that cortisol responders displayed worse mood and higher restlessness after stress induction compared to controls (both *p* < 0.001) but not compared to nonresponders (both *p* > 0.27) (Figure 3).

### Decision making

#### Test of working memory as covariate

Groups did not differ regarding working memory performance (*p* > 0.11). Mean performance was 9.25 ± 2.14 points (it was maximally possible to reach 14 points). A repeated measures ANCOVA on risk taking was performed to test working memory performance as potential covariate. A significant main effect (*p* = 0.041) and interaction with expected value (*p* = 0.033) were obtained. Therefore, working memory performance was included as covariate in further analyses of risk taking. For the ambiguity part, working memory performance was not relevant as covariate (*p* > 0.12).

#### Risk

First, we investigated whether the stress group as a whole differed from the control group regarding decision making in risky lotteries and whether such an effect was moderated by the domain of decision making. A repeated measures ANCOVA including working memory performance as covariate was conducted, with domain (gain, loss, and mixed) and expected value (higher, equal, lower) as repeated factors, and group (stress, control) and gender as between subject factors. No significant main or interaction effects for group were obtained (all *p* > 0.16). Next, we repeated the analysis with the divided stress group, i.e., comparing cortisol responders, nonresponders, and controls. A significant main effect of group emerged [*F*_(2, 68)_ = 3.36, *p* = 0.040]. Contrasts indicated that risk taking was higher in responders compared to controls (*p* = 0.026; percentage of risky choices: 0.49 ± 0.17 vs. 0.41 ± 0.13) as well as in comparison to nonresponders (*p* = 0.031; 0.43 ± 0.14). Nevertheless, as the triple interaction between group × domain × expected value was tentatively significant as well [*F*_(7.81,265.70)_ = 1.95; *p* = 0.055], we repeated the analysis for each domain separately.

**Figure 4:**
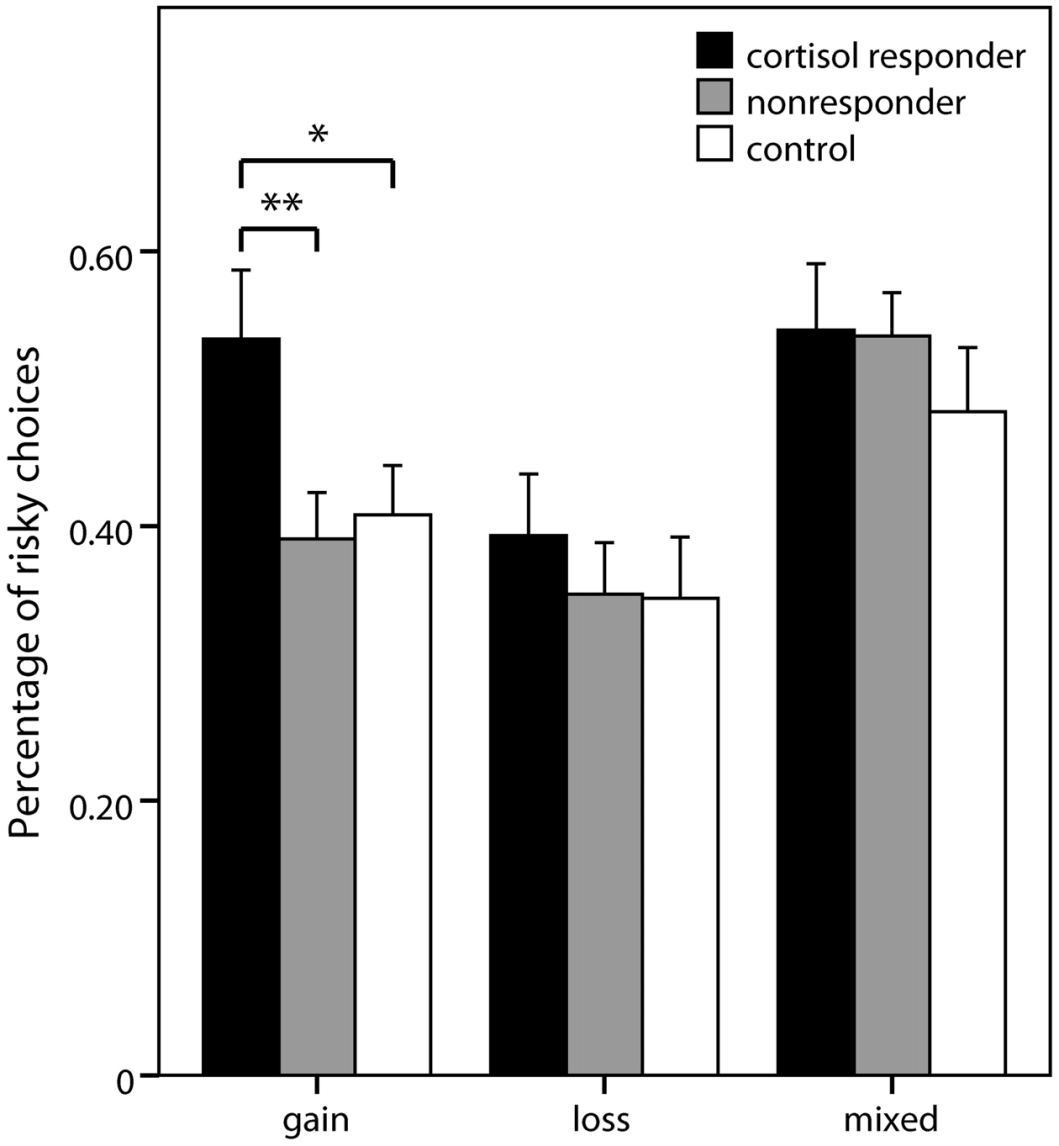
**Risky choices in the decision task**. Percentage (± s.e.m.) of choices of the risky option (vs. sure amounts) in the three domains of the risk part of the decision making task. Simple contrasts comparing cortisol responders to nonresponders and controls were used to follow up significant main effects (^*^p < 0.05, ^**^p < 0.01).

In the pure gain domain, a significant main effect of group was again obtained [*F*_(2, 68)_ = 5.87; *p* = 0.004] with contrasts showing that responders chose more risky lotteries than controls (*p* = 0.011; percentage of risky choices: 0.54 ± 0.26 vs. 0.41 ± 0.16) and nonresponders (*p* = 0.002; 0.39 ± 0.18). No significant group differences were obtained in the analyses of pure loss and mixed domains (Figure 4).

#### Ambiguity

**Figure 5:**
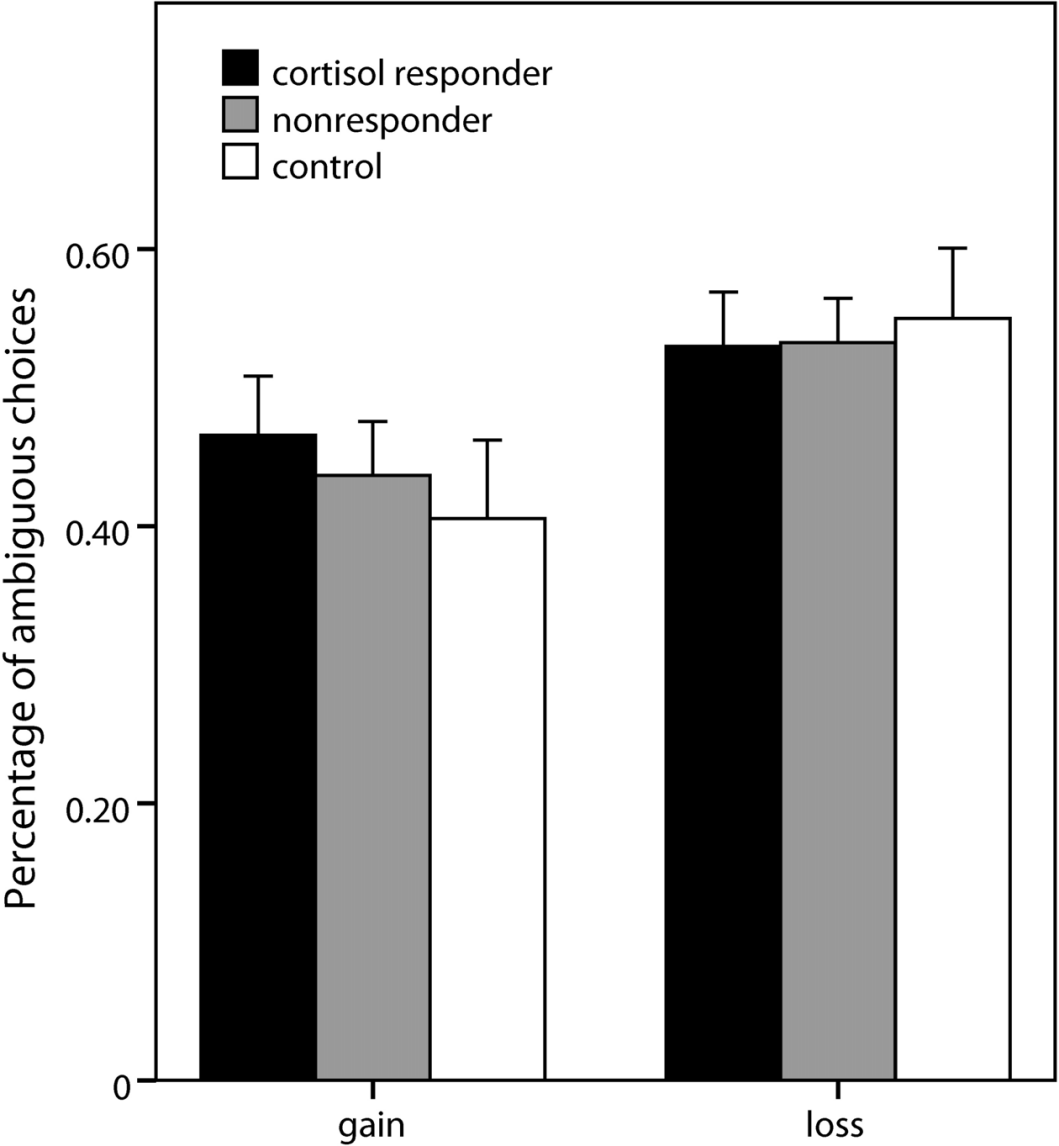
**Ambiguous choices in the decision task**. Percentage (±s.e.m.) of choices of the ambiguous option (vs. risky lotteries) in the two domains of the ambiguity part of the decision making task.

Analogous to the risk part, a 2 (domain) × 3 (expected value) × 2 (group) × 2 (gender) ANOVA was conducted, comparing the stress group as a whole with the control group^5^. No significant effects of group or gender were observed. Also, when the analysis was repeated comparing cortisol responders (percentage of ambiguous choices: 0.50 ± 0.18), nonresponders (0.49 ± 0.17), and controls (0.48 ± 0.23), no significant effect of group was obtained (Figure 5). Significant main effects of domain [*F*_(1, 69)_ = 18.16, *p* < 0.001] and expected value [*F*_(2, 138)_ = 96.64, *p* < 0.001] as well as a significant interaction of domain and expected value [*F*_(2, 138)_ = 4.94, *p* = 0.008] emerged, pointing to an expected value-dependent reflection effect, i.e., higher ambiguity acceptance for losses than for gains specifically for higher expected values of the ambiguous alternative.

## Discussion

In this study, we investigated whether acute psychosocial stress affects decision making under uncertainty, i.e., under risk and under ambiguity, in a set of binary lotteries associated with real monetary incentives. We varied decision domain (i.e., gain vs. loss vs. mixed) and expected value of the risky alternative systematically, and did not provide any feedback between the choices—with the aim of investigating risk taking independent of learning processes. Those participants that were strongly affected by the stressor as indicated by a cortisol increase of at least 2.5 nmol/l showed considerably more risk taking in the pure gain domain compared to controls.

### Stress affects risk taking independent of feedback

Previous studies investigating stress effects on decision making mostly used tasks that included direct feedback of the outcome after each trial (Preston et al., 2007; Lighthall et al., 2009, 2012; Porcelli and Delgado, 2009; van den Bos et al., 2009; Pabst et al., 2013a,b,c; Gathmann et al., 2014) and, therefore, the observed stress effects on decision making could not be teased apart from stress effects on learning from feedback. Indeed, several studies found stress effects on feedback learning in other fields than decision making (Petzold et al., 2010; Lighthall et al., 2013). Only one study implemented a no-feedback condition in the decision making task and still observed a trend for more risk taking (Starcke et al., 2008), thus pointing to the possibility that decision making under uncertainty *per se* could be affected by stress. Applying a task containing no feedback, our results strongly support this presumption. Furthermore, our subjects were highly motivated to perform advantageously in the task as part of their study payment depended on their choices. As our payment scheme of randomly selecting trials for payment discourages additional processes such as mental accounting, we assume that the observed differences in risk taking truly reflect stress-dependent influences on risk preferences.

### Domain specificity of the stress effect

We observed a higher rate of risk taking of cortisol responders only in the gain domain. This is in contrast to the results of Porcelli and Delgado (2009) and Pabst et al. (2013b) who also found evidence for domain-specific modulation of risk taking. Yet, whereas Porcelli and Delgado (2009) report more risk taking in the loss domain and less risk taking in the gain domain, Pabst et al. (2013b) found less risk taking specifically in the loss domain. Besides the different types of stressors used and missing information about cortisol responses in the study by Porcelli and Delgado (2009), there are important differences regarding task characteristics that could potentially account for the contradicting results. First of all, choice was between two risky alternatives of varied probability in Porcelli and Delgado (2009) whereas in our task one option was a risky lottery and the other one a sure amount. Similarly, in Pabst et al. (2013b) who applied a modified version of the GDT, choice was between 14 risky options. Furthermore, participants were not paid according to their choices in Pabst et al. (2013b). Most importantly, the feedback provided in the studies of Porcelli and Delgado (2009) and Pabst et al. (2013b) might have influenced subsequent choice behavior.

Our results further seem to be at odds with a recent study investigating the effect of anticipatory stress on a probability discounting task embedded in a delay discounting paradigm (Lempert et al., 2012). Here, choices had to be made between varying sure amounts and lotteries in the gain domain at several probability levels until indifference points were established. Importantly, no immediate feedback was provided. In this study, no stress effects on risk taking were observed and the correlation between cortisol and risk taking was nonsignificant as well. Again, we consider the possibility that differences regarding the stress induction protocols or specific task characteristics might be responsible for these contradicting results. One important issue might be that the reported average cortisol response was very low in the study of Lempert et al. (2012) and probably comprised mainly subjects that we would have classified as nonresponders.

Contrary to our expectations based on the results of previous studies that reported a higher rate of risk taking in tasks that included the mixed domain (Starcke et al., 2008; Pabst et al., 2013c), we did not observe significantly more risk taking in the mixed domain. There are several plausible reasons for this difference. One possibility is that different cognitive processes might be involved in tasks containing losses that are differentially affected by stress compared to tasks with gains only. Therefore, task characteristics like the complete randomization, i.e., mixture of domains in the present study, might have affected our results. Alternatively, and as already discussed above, disturbed learning from feedback might be the mechanism behind the higher risk proneness reported for mixed gambles so far. The study of Starcke et al. (2008) which included one condition with and one without feedback in a mixed domain gamble supports these interpretations as the stress effect was significant in the feedback condition while it diminished without feedback. Mixed gambles might be more difficult to process as gain and loss amounts and their respective probabilities have to be integrated. Therefore, feedback about previous decision outcomes might be an important mechanism influencing choice in mixed gambles.

### Differential stress effects on risk vs. ambiguity

The present study is, to the best of our knowledge, the first study that investigated stress effects on decision making under ambiguity independent from feedback learning. We did not find evidence for altered decision making under ambiguity. It is important to keep in mind that the alternative option in this task was a risky prospect. Our results therefore indicate that stress does not increase the propensity to accept uncertainty beyond that of specified risks. Once again, learning from feedback might be the driving mechanism behind the stress effects reported for decision making under ambiguity in previous studies (i.e., Preston et al., 2007; Lighthall et al., 2009; van den Bos et al., 2009). Our results furthermore point to the importance of distinguishing between decisions from description and decisions from experience when investigating stress effects on economic decision making.

### Role of cortisol in mediating the stress effects

In the present study, we found no differences in gambling behavior between the stress group as a whole and controls. High variability in the response to stress regarding physiological stress markers was noted earlier (e.g., Kudielka et al., 2009), but also regarding stress effects on decision making (Starcke et al., 2008). Thus, two reasonable explanations exist why we found stress effects on decision making only in the subgroup of cortisol responders. If the cortisol increase is taken as an objective indicator of experienced stressfulness, only those participants that were reliably affected by our stress induction protocol showed stress effects on decision making. On the other hand, cortisol could itself be mechanistically involved in the observed effect. Previous studies suggest a causal role for cortisol in mediating stress effects on risk taking (Starcke et al., 2008; van den Bos et al., 2009; Pabst et al., 2013a), although this might not be the only mechanism behind stress effects (Lighthall et al., 2012). Indeed, recent pharmacological studies reported effects of hydrocortisone administration on risk taking in lotteries (Putman et al., 2010; Kandasamy et al., 2014).

One possible molecular mechanism could be the interplay of stress-induced supraoptimal levels of cortisol and dopamine in the prefrontal cortex (PFC), restraining functioning of this brain region that is known to exert top-down control over behavior. Stress leads to a shift from prefrontal to subcortical processing of information, resulting in loss of top-down control and strengthening of stimulus-driven reactions (Piazza and Le Moal, 1997; Erickson et al., 2003; Arnsten, 2009; Scholz et al., 2009; Schwabe et al., 2012). Stimuli that are attractive at first glance, like the possibility to win, could then override cognitive control mechanisms and more directly influence behavior, leading to a higher probability of choosing risky options (cf. Knoch et al., 2006). A recent fMRI study indeed pointed to decreased prefrontal activity during processing of rewards in stressed subjects (Ossewaarde et al., 2011). Similar mechanisms are also suggested by studies using stimulation techniques (i.e., repetitive transcranial magnetic stimulation and transcranial direct current stimulation) to directly manipulate PFC functioning during risky decision making (Knoch et al., 2006; Fecteau et al., 2007a,b).

Additionally, stress could lead to increased dopaminergic signaling in the striatum, thereby increasing reward salience and risk taking (cf. Mather and Lighthall, 2012; Starcke and Brand, 2012). Indeed, there is evidence for increased dopaminergic activity in striatal regions under stress, which might be mediated by glucocorticoids (Piazza and Le Moal, 1997; Adler et al., 2000; Pruessner et al., 2004; Scott et al., 2006). Imaging as well as pharmacological studies substantiate the involvement of the striatum and the dopaminergic system in reward anticipation (Knutson et al., 2005; Kuhnen and Knutson, 2005; Trepel et al., 2005; Rogers, 2011; St. Onge et al., 2011). Furthermore, altered decision making is reported for patients with perturbed dopaminergic system functioning and in relation to several dopaminergic polymorphisms (for a recent review, see Rogers, 2011).

### Limitations

It could be seen as a limitation that a between-subjects design was used in the study. Although this is a common way to investigate stress effects on cognitive performance, a within-subjects design would be valuable in order to reduce heterogeneity regarding risk preferences as well as stress responses. To address this issue, working memory was assessed as covariate in our study, but only after the stress induction and the decision making task. Nevertheless, as group means did not differ regarding this measure, we assume that stress has not influenced it. Furthermore, stress seems not to affect working memory as assessed with simple tasks like the digit span forward task that was used in our study (Schoofs et al., 2009). Another limitation concerns the presentation format of the risk task. Probabilities were depicted graphically, so there might have been some vagueness regarding the actual numerical probabilities. Future studies should provide the probabilities in a numerical format. It should be mentioned that the division of the stress group into cortisol responders and nonresponders, although common in stress research, leads to a correlational nature of data. It cannot be excluded that a third factor influences both, risk taking and the cortisol response to stress, or that those who are risk seeking are those who show a marked response to acute stress. Yet, in our opinion causal influences of cortisol on risk taking are the most plausible (see Discussion).

## Conclusion

Our study shows that stress, as indicated by a reliable cortisol response, can affect risk taking independent of feedback-based learning processes. Yet, this effect seems to be highly dependent on task characteristics. Here, we observed a higher rate of risk taking in cortisol responders only in the pure gain domain but not in the pure loss and the mixed domains. Furthermore, acute stress did not influence gambling under ambiguity. More generally, our results point to the importance of considering crucial distinctions made by economic theory when investigating stress effects on economic decision making.

### Conflict of interest statement

The authors declare that the research was conducted in the absence of any commercial or financial relationships that could be construed as a potential conflict of interest.

## Footnotes

^1^Note that Knight (1921) used the term uncertainty instead of the term ambiguity which was introduced by Ellsberg (1961) and is commonly used today. Throughout the paper we use uncertainty as an umbrella term.

^2^exception: L-Thyroxin (1 participant) was allowed.

^3^*N* = 74 as one participant did not provide this information.

^4^Furthermore, risk preferences were assessed using the paper-and-pencil version of a widely used multiple price list (Holt and Laury, 2002). Yet, as this task did not turn out to be relevant as covariate (probably because it differs in several respects from the risk task applied under stress) it was not included in the analyses and will not be considered in detail here.

^5^Note that for the analysis of decision making behavior in the risk and ambiguity part repeated measures ANOVAs were applied, assuming a metric and normally distributed variable. Applying an approach that does not make these assumptions, i.e., generalized estimating equations, we reach the same conclusions for both parts of the task.
